# Evolution of endovascular repair of complex aortic aneurysms in a German tertiary referral vascular center

**DOI:** 10.3389/fsurg.2026.1743595

**Published:** 2026-02-06

**Authors:** Yannik Wanner, Shamsun Naher, Maria Del Pilar Ortega Carrillo, Michael Kallmayer, Felix Kirchhoff, Matthias Trenner, Christoph Knappich

**Affiliations:** 1 Department of Vascular and Endovascular Surgery, Munich Aortic Center, TUM University Hospital, Klinikum Rechts der Isar, Technical University of Munich, Munich, Germany; 2 Division of Vascular Medicine, St.-Josefs Hospital, Wiesbaden, Germany

**Keywords:** branched endovascular aortic repair (bEVAR), complex aortic aneurysms, fenestrated endovascular aortic repair (FEVAR), thoracoabdominal aortic aneurysms, juxtarenal aortic aneurysms

## Abstract

**Background:**

Fenestrated and/or branched endovascular aortic repair (f/bEVAR) has evolved a reliable alternative to treat complex aortic aneurysms. The aim of this study was to analyze the evolution of f/bEVAR in a large German vascular department by investigating temporal changes in patient selection, surgical strategies, and clinical outcomes.

**Methods:**

Retrospective cohort study of consecutive patients undergoing f/bEVAR between 2007 and 2023 at TUM University Hospital in Munich (Klinikum rechts der Isar, Technical University of Munich). To assess for temporal changes, the cohort was divided into three time periods (date of operation: 2007–2016; 2017–2020; 2021–2023). The primary outcome was in-hospital death. Statistical analyses included univariate analyses, Kaplan–Meier survival analyses, and Kruskal–Wallis tests for group comparisons.

**Results:**

A total of 176 patients (median age 75 years; 80% male) were included. Over time (*early phase* vs. *recent phase*), an increase in the proportion of octogenarians was observed (14 vs. 31%; *p* = 0.042) and the mean number of incorporated vessels increased from 3.7 to 4.0 (*p* < 0.001). Mean operative time decreased from 321 to 241 min (*p* = 0.002) and intraoperative contrast volume was reduced (398 vs. 190 mL; *p* = 0.001). Length of intensive care unit stay (8.1 vs. 2.7 days; *p* < 0.001) and in-hospital rates of acute kidney injury (16 vs. 4.7%; *p* = 0.034) and respiratory failure (18 vs. 0%; *p* = 0.001) declined, while non-significant trends were found for in-hospital mortality (8.8% vs. 1.6%; *p* = 0.062) and the paraplegia rate (8.8% vs. 1.6%, *p* = 0.062). Longer operating time (per 10 min; OR 1.06; 95% CI 1.02–1.11; *p* = 0.003) and occurrence of a major adverse event (OR 37.4; 95% CI 4.52–4,869; *p* < 0.001) were associated with death until discharge. Kaplan–Meier analyses showed, that patients treated in the *early phase* had lower survival probability compared to those in the *recent phase* (*p* = 0.024).

**Conclusion:**

This retrospective analysis demonstrates a continuous improvement in clinical outcomes associated with f/bEVAR over the past two decades. The findings underscore the increasing reliability and effectiveness of endovascular treatment approaches.

## Introduction

Complex aortic aneurysms constitute a heterogenous group of conditions including juxta-, pararenal, and thoracoabdominal aortic aneurysms (TAAA) and are characterized by profound variability on morphological, histological, and etiological levels. The natural course of disease implying a risk of aneurysm rupture, as well as surgical treatment are associated with significant morbidity and mortality.

Current international guidelines recommend surgical treatment of TAAAs if the diameter has reached a threshold of 6 cm (1–4).

Due to a lack of alternative treatment options, open surgical repair (OSR) was the standard of care for many decades. However, the substantial surgical access trauma, clamping and de-clamping of the aorta including kidneys and abdominal organs, and blood loss, imposed a relevant impact on a patient's organism, translating into high mortality (>10%) and morbidity (e.g., cardiac events, acute kidney injury, bowel ischemia, spinal cord ischemia) (5). Over time, the implementation of adjunctive techniques such as extracorporeal circulation with distal perfusion and selective perfusion of arterial branches, intercostal artery reimplantation, cerebrospinal fluid (CSF) drainage, pre-operative minimally invasive selective segmental artery coil-embolization (MISACE), and peri-operative assessment of spinal cord function have contributed to an improvement of outcomes (6, 7). However, the most incisive change in treatment of TAAA was heralded by the evolution of endovascular aortic repair (EVAR) techniques. While those concentrated on pathologies of the infrarenal aorta in the early 1990ies (8), further developments with the implementation of fenestrations, scallops, and branches with different configurations have provided the opportunity to treat most complex aortic aneurysms.

The aim of this study was to characterize the evolution of fenestrated and/or branched EVAR (f/bEVAR) to treat complex aortic aneurysms in a large German vascular center and the associated changes of outcomes. Furthermore, potential risk markers were to be defined.

## Methods

### Study design

A retrospective descriptive analysis of consecutive f/bEVAR procedures to treat complex aortic aneurysms between March 2007 and December 2023 was performed. Patients were identified from a prospectively maintained aortic database of the Department of Vascular and Endovascular Surgery at TUM University Hospital (Klinikum rechts der Isar, Technical University of Munich). The study was conducted following the Strobe Statement (9). The Ethics Committee of the University Hospital of the Technical University of Munich approved the data collection under the reference number 2025-194-S-KK. The analysis follows the predefined reporting standard for endovascular aortic repair of aneurysms involving the renal-mesenteric arteries (10).

### Patients and procedures

**Figure 1:**
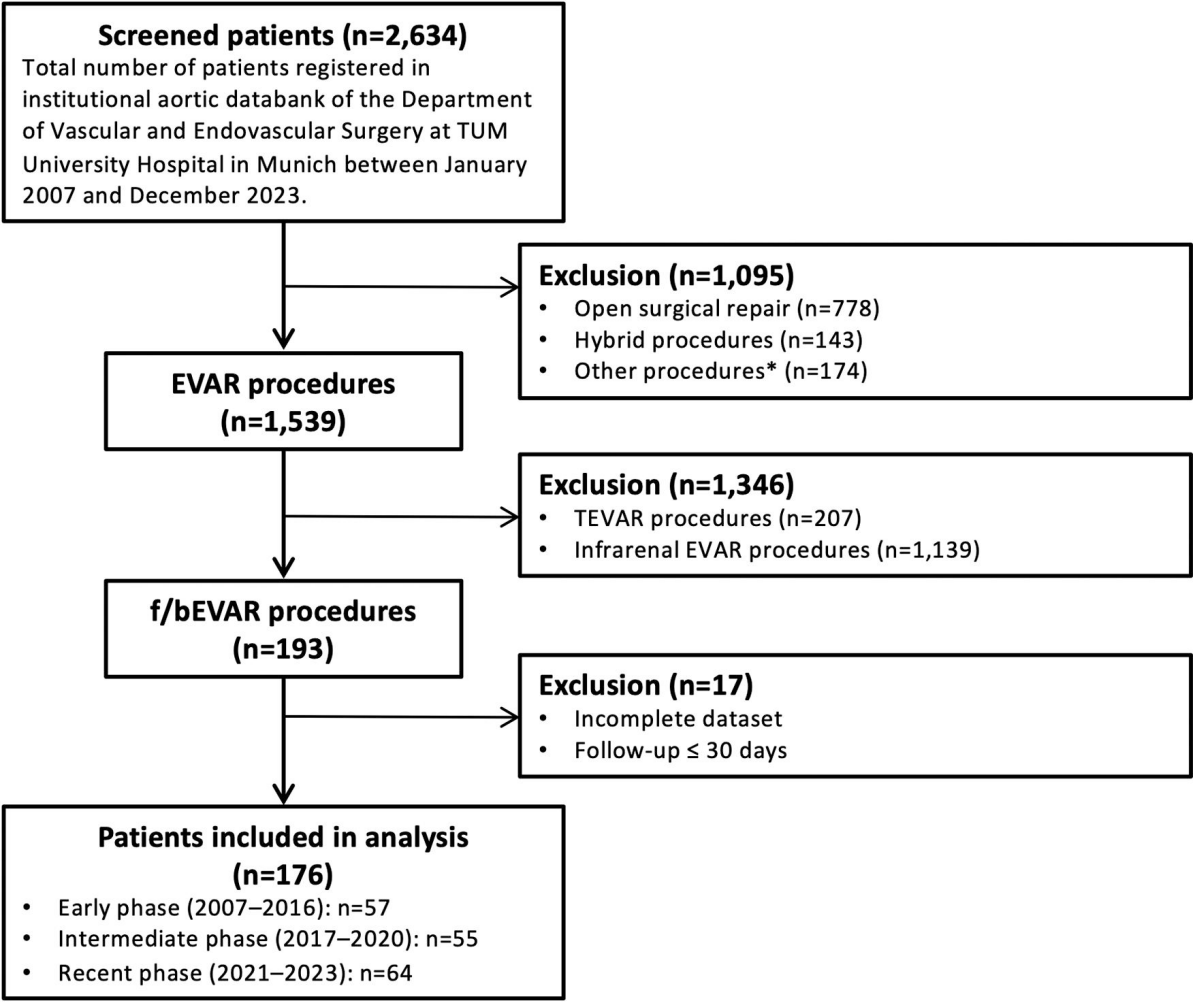
Patient flowchart. TUM, Technical University of Munich; EVAR, Endovascular Aortic Repair; TEVAR, Thoracic Endovascular Aortic Repair; f/bEVAR, fenestrated and/or branched Endovascular Aortic Repair; * open surgical or endovascular procedures to treat endoleaks, graft migrations, aortic occlusions, (graft) infections.

Patients were included, if they underwent f/bEVAR during the study period (Figure 1). Treated pathologies included TAAAs (classified according to modified Crawford classification) (11), juxta/pararenal abdominal aortic aneurysms (j/pAAA), and/or aortic dissections. Decisions on indications for repair were achieved in a multidisciplinary team meeting and based on international guidelines (1). TAAAs and j/pAAAs were usually operated, if they reached a threshold diameter of at least 60 mm, showed rapid growth (≥5 mm/6months or ≥10 mm/12 months), were symptomatic or ruptured, or featured penetrating aortic ulcer (PAU) morphology. All included aortic dissections showed chronic degenerative expansions. Decisions whether to perform open surgical repair or f/bEVAR based on patients’ preference, patients’ age and comorbidities, and feasibility. If f/bEVAR was performed, a percutaneous access was performed as standard approach. For transbrachial implantation of bridging-stents, an open surgical access close to the axilla was conducted. During the procedure, patients were fully heparinized with continuous heparin infusion aiming for an activated clotting time (ACT) of 250–280 s. Stent grafts were chosen based on patients’ anatomy, availability (in urgent or emergency cases), and surgeons’ preference. Spinal cord ischemia (SCI) prevention strategies considered extent and location of the aortic pathology, patency of vertebral arteries and internal iliac arteries, existence, number, and diameter of intercostal/lumbar arteries. If the risk for SCI was considered as relevant, staged repair (if feasible) or preemptive spinal drainage were performed. During and after the operation, it was aimed to achieve a mean arterial pressure (MAP) ≥90 mmHg, a hemoglobin value ≥9 mg/dL, and oxygen was applied through a nasal cannula after extubation. If the patient presented with signs of SCI and no spinal drain had been applied beforehand, it was established urgently. Especially towards the end of the study period, the mentioned postoperative measures were adhered to meticulously and if a spinal drain had been placed, it was removed not before the third postoperative day. If a staged repair was performed, usually one branch was used as perfusion branch. The completion procedure was performed at the earliest 6 weeks after the index procedure. In the second half of the study period, we aimed to perform the completion procedure under local anesthesia, blocked the branch with a balloon catheter for 15 min, and only implanted the bridging stent if the patient remained asymptomatic. The first f/bEVAR procedures were performed with a mobile C-arm (Veradius, Philips Medical Systems, Eindhoven, Netherlands or OEC 9800, GE Healthcare, Chicago, USA), from 2013 all procedures were performed in a hybrid operating room (2013–2017: Allura Xper FD20, Philips Medical Systems, Eindhoven, Netherlands; 2017–2023: Azurion 7 C20 with FlexArm, Philips Medical Systems, Eindhoven, Netherlands). The follow-up (FU) algorithm in our department comprises routine follow-up visits with contrast enhanced ultrasound (CEUS) scans at 1, 6, and 12 months after the index procedure. Thereafter, follow-up is conducted annually. Computed tomography angiography (CTA) scans are usually performed within one month after the procedure and annually thereafter. (Endovascular) repair is indicated, if a high-flow endoleak (type I or III) or a low-flow endoleak (type II) with relevant aneurysm growth (≥5 mm in diameter) is detected.

### Data collection

Clinical patient data during the hospital stay, including pre-operative and operative characteristics and complications, and FU data were obtained from electronic medical records. FU intervals and modalities varied according to clinical practice.

### Outcomes

The primary outcome was defined as in-hospital death after the index procedure. Secondary outcomes were death within 30 days after the index procedure and death during the FU. Other outcomes included length of intensive care unit (ICU) stay, length of hospital stay, any secondary intervention, aortic secondary intervention, occurrence of any component or a combination of major adverse events [MAE; comprising blood loss >1,000 mL, myocardial infarction, acute kidney injury (i.e., >50% decrease in glomerular filtration rate), respiratory failure, paraplegia, stroke, bowel ischemia], and evidence of an endoleak in post-operative CTA scans.

### Statistical analyses

Categorical variables were presented as absolute numbers and percentages. Continuous data were presented as median and interquartile range if not normally distributed. Normally distributed interval scaled data were presented as mean and standard deviation. To assess temporal trends in treatment and outcomes, the cohort was divided into three equal treatment periods: early phase (2007–2016), intermediate phase (2017–2020), and recent phase (2021–2023). Time intervals were defined *a priori* after a first assessment of data in order to contain similar numbers of patients. As a certain number of patients was excluded from the final analyses (Figure 1), the final number of analyzable patients per period varied slightly.

Univariate analyses were used to assess for clinically associated risk factors for the occurrence of in-hospital death. Survival analyses were performed using the Kaplan–Meier method with log-rank testing. Group comparisons were made using Kruskal–Wallis and chi-square tests as appropriate. A *p*-value < 0.05 was considered statistically significant. Statistical analyses were performed using R Studio, version 4.0.3 (R Foundation for Statistical Computing, Vienna, Austria).

## Results

A total of 176 patients undergoing f/bEVAR between 2007 and 2023 were included in the study. 57 of them were treated in the early phase (before 2017), while 55 and 64 patients were treated in the intermediate (between 2017 and 2020) and the recent phase (after 2020).

### Demographics and clinical characteristics

The median age of the population was 75 years with 80% being male (Table 1). The proportion of octogenarians increased from 14% in the early phase to 31% in the recent phase (*p* = 0.022).

**Table 1:** Demographics and clinical characteristics.

Variable	Overall (*n* = 176)	Early phase (<2017) (*n* = 57)	Intermediate phase (*n* = 55)	Recent phase (>2020) (*n* = 64)	*p*-value
Demographics					
Age in years (median; Q1–Q3)	75 (70–80)	74 (68–79)	75 (70–80)	78 (72–81)	0.075
Age > 80 years	39 (22)	8 (14)	11 (20)	20 (31)	**0**.**022**
Male	140 (80)	47 (82)	44 (80)	49 (77)	0.421
Emergency procedure	23 (13)	5 (8.8)	9 (16)	9 (14)	0.405
AAA characteristics					
Aneurysm size in mm (median; Q1–Q3)	59 (54–65)	58 (53–62)	58 (54–64)	59 (55–67)	0.572
Prior aortic repair	41 (23)	11 (19)	11 (20)	19 (30)	0.170
Aortic dissection	5 (2.8)	1 (1.8)	1 (1.8)	3 (4.7)	0.324
Aneurysm type					
Crawford Type I	10 (5.7)	3 (5.3)	3 (5.5)	4 (6.3)	0.486
Crawford Type II	10 (5.7)	5 (8.8)	2 (3.6)	3 (4.7)	
Crawford Type III	14 (8.0)	7 (12)	5 (9.1)	2 (3.1)	
Crawford Type IV	15 (8.5)	5 (8.8)	5 (9.1)	5 (7.8)	
Crawford Type V	3 (1.7)	0 (0.0)	2 (3.6)	1 (1.6)	
Juxta/ pararenal	119 (68)	36 (63)	37 (67)	46 (72)	
Comorbidities					
Hypertension	171 (97)	53 (93)	55 (100)	63 (98)	0.080
Coronary artery disease	93 (53)	33 (58)	25 (45)	35 (55)	0.757
Congestive heart failure	33 (19)	12 (21)	7 (13)	14 (22)	0.873
Arrhythmia	40 (23)	13 (23)	8 (15)	19 (30)	0.338
PAD	39 (22)	14 (25)	9 (16)	16 (25)	0.922
Stroke/TIA	36 (20)	11 (19)	12 (22)	13 (20)	0.088
COPD	33 (19)	15 (26)	9 (16)	9 (14)	0.195
Cigarette smoking					
Ex-smoker	93 (53)	28 (49)	35 (64)	30 (47)	0.140
Active smoker	40 (23)	18 (32)	9 (16)	13 (20)	
Cancer	46 (26)	15 (26)	14 (25)	17 (27)	0.972
Diabetes mellitus	37 (21)	13 (23)	9 (16)	15 (23)	0.906
Hypercholesterolemia	150 (85)	42 (74)	49 (89)	59 (92)	**0**.**005**
CKD III-V	22 (13)	8 (14)	6 (11)	8 (13)	0.810
Medication					
Anticoagulation	40 (23)	11 (19)	8 (15)	21 (33)	0.068
Platelet aggregation inhibitor(s)	172 (98)	54 (95)	55 (100)	63 (98)	0.187
Antihypertensive(s)	164 (93)	49 (86)	53 (96)	62 (97)	**0**.**019**
Statin	142 (81)	36 (63)	48 (87)	58 (91)	**<0**.**001**
ASA Classification					
II	34 (19)	15 (26)	6 (11)	13 (20)	0.718
III	129 (73)	39 (68)	44 (80)	46 (72)	
IV	13 (7.4)	3 (5.3)	5 (9.1)	5 (7.8)	

Values given as *n* (%) unless otherwise stated. n, number of patients; Q1, first quartile; Q3, third quartile; PAD, peripheral artery disease; TIA, transitory ischemic attack; COPD, chronic obstructive pulmonary disease; CKD, chronic kidney disease; ASA, American Society of Anesthesiologists.

Bold font indicates statistical significance.

In 13% of cases, f/bEVAR was performed as an emergency procedure. The median diameter of the aortic aneurysm amounted to 59 mm. Regarding aneurysm type, patients with juxta/pararenal aneurysms represented the largest cohort (68%), followed by TAAAs type IV (8.5%), type III (8.0%), type I and II (5.7% each), and type V (1.7%) according to the modified Crawford classification (11).

Comorbidities included hypertension (97%), coronary artery disease (53%), peripheral arterial occlusive disease (22%), stroke or transient ischemic attack (20%), chronic obstructive pulmonary disease (COPD; 19%), diabetes mellitus (21%), and hypercholesterolaemia (85%).

Most patients ranged in American Society of Anaesthesiologists (ASA) stage III (73%), followed by stages II (19%) and IV (7.4%).

Medical treatment included antiplatelet agents (98%), anticoagulants (23%), antihypertensives (93%), and statins (81%). Statin use significantly increased across treatment phases (*p* < 0.001).

### Device design

The majority of patients (71%) were treated with custom made devices, while 26% underwent treatment with an off-the-shelf device. In 2.8%, a physician modified endograft (PMEG) was implanted (Table 2).

**Table 2:** Device design.

Variable	Overall (*n* = 176)	Early phase (<2017) (*n* = 57)	Intermediate phase (*n* = 55)	Recent phase (>2020) (*n* = 64)	*p*-value
Custom made device	125 (71)	45 (79)	37 (67)	43 (67)	0.162
Cook	82 (47)	43 (75)	23 (42)	16 (25)	**<0**.**001**
FEVAR^a^	35 (20)	17 (30)	8 (15)	10 (16)	
FEVAR w/scallop^b^	34 (19)	21 (37)	11 (20)	2 (3.1)	
bEVAR	6 (3.4)	3 (5.3)	2 (3.6)	1 (1.6)	
f/bEVAR	7 (3.9)	2 (3.5)	2 (3.6)	3 (4.7)	
Terumo aortic (Vascutec) fenestrated anaconda	39 (22)	1 (1.8)	12 (22)	26 (41)	**<0**.**001**
Terumo fenestrated treo	1 (0.6)	0 (0.0)	0 (0.0)	1 (1.6)	0.245
Artivion (Jotec) E-xtra	3 (1.7)	1 (1.8)	2 (3.6)	0 (0.0)	0.431
Not custom made device	46 (26)	12 (21)	15 (27)	19 (30)	0.284
Cook t-Branch	43 (24)	12 (21)	15 (27)	16 (25)	0.628
Artivion (Jotec) E-nside	3 (1.7)	0 (0.0)	0 (0.0)	3 (4.7)	**0**.**043**
Physician modified endograft	5 (2.8)	0 (0.0)	3 (5.5)	2 (3.1)	0.324
Vessels per patient (mean; SD)	3.8 (0.6)	3.7 (0.5)	3.5 (0.7)	4.0 (0.4)	**<0**.**001**
≥4 vessels	142 (81)	44 (77)	37 (67)	61 (95)	**0**.**009**
Fenestrations					
Celiac axis	69 (39)	16 (28)	16 (29)	37 (58)	**0**.**001**
SMA	96 (55)	34 (60)	20 (36)	42 (66)	0.445
Renal arteries	122 (69)	43 (75)	34 (62)	45 (70)	0.574
Directional branches					
Celiac axis	59 (34)	15 (26)	22 (40)	22 (34)	0.370
SMA	56 (32)	14 (25)	22 (40)	20 (31)	0.461
Renal arteries	52 (30)	14 (25)	19 (35)	19 (30)	0.559
Scallops					
Celiac axis	21 (12)	14 (25)	4 (7.3)	3 (4.7)	**0**.**001**
SMA	16 (9.1)	8 (14)	7 (13)	1 (1.6)	**0**.**016**
Iliac branch device	4 (2.3)	1 (1.8)	0 (0.0)	3 (4.7)	0.261
Cervical debranching	2 (1.1)	1 (1.8)	0 (0.0)	1 (1.6)	0.946
Conduit	4 (2.3	1 (1.8)	2 (3.6)	1 (1.6)	0.923

Values given as *n* (%) unless otherwise stated. n, number of patients; FEVAR, fenestrated endovascular aortic repair; w/, with; bEVAR, branched endovascular aortic repair; SD, standard deviation; SMA, superior mesenteric artery.

a Contains endografts with 1 (*n* = 1), 2 (*n* = 3), 3 (*n* = 2), 4 (*n* = 28), and 5 (*n* = 1) fenestrations.

b Contains endografts with 1 (*n* = 2), 2 (*n* = 14), and 3 (*n* = 18) fenestrations.

Bold font indicates statistical significance.

The number of incorporated vessels increased from an average of 3.7 in the early phase to 4.0 in the recent phase (*p* < 0.001). Accordingly, the proportion of patients receiving fenestrations for the celiac axis increased from 26% to 34% (*p* = 0.001) comparing the early and recent phases. Contrarily, a decline of scallops for the celiac axis (25 vs. 4.7%) and the superior mesenteric artery (SMA; 14 vs. 1.6%) was observed.

Regarding types of implanted devices, custom made devices (CMD) manufactured by Cook (Cook Medical, Bloomington, USA) were implanted most frequently (47%), followed by the off-the-shelf t-Branch endograft (24%; Cook Medical, Bloomington, USA) and the fenestrated Anaconda (22%; Vascutek/Terumo Aortic, Inchinnan, UK). While fenestrations were frequently combined with scallops (37% of all devices) in the early phase, only 3.1% of patients in the recent phase were treated with a combination of fenestrations and scallop. The proportion of purely fenestrated endografts manufactured by Cook declined from 30% in the early phase to 16% in the recent phase. Conversely, the proportion of fenestrated Anaconda endografts rose from 1.8% in the early phase to 41% in the recent phase. Regarding the t-Branch endograft, the implantation habit remained stable over time.

Overall, f/bEVAR was combined with iliac branch devices (IBD) in 2.3% and with cervical debranching procedures in 1.1%.

### Procedural details

All operations were performed under general anesthesia (Table 3). Cerebrospinal fluid drainage was applied in a third of the patients and in 99% of the patients a percutaneous access was used. Brachial access was performed in one third of the patients with a non-significant decline comparing early and recent phases (39 vs. 23%; *p* = 0.072).

**Table 3:** Procedural details.

Variable	Overall (*n* = 176)	Early phase (<2017) (*n* = 57)	Intermediate phase (*n* = 55)	Recent phase (>2020) (*n* = 64)	*p*-value
General anesthesia	176 (100)	57 (100)	55 (100)	64 (100)	–
Cerebrospinal fluid drainage	58 (33)	19 (33)	20 (36)	19 (30)	0.655
Percutaneous femoral approach	174 (99)	57 (100)	54 (98)	63 (98)	0.429
Brachial access	58 (33)	22 (39)	21 (38)	15 (23)	0.072
Staged repair	28 (16)	10 (18)	6 (11)	12 (19)	0.825
Time between main and final operation in days (mean; SD)	96 (139)	185 (220)	53 (33)	51 (26)	0.078
Operating time in minutes (mean; SD)	274 (131)	321 (154)	265 (118)	241 (108)	**0**.**002**
Amount of contrast used in mL (mean; SD)	257 (676)	398 (1,165)	187 (106)	190 (85)	**0**.**001**
Total fluoroscopy time in minutes (mean; SD)	65 (33)	63 (36)	61 (32)	68 (33)	0.281
Estimated blood loss in mL (mean; SD)	874 (1,032)	965 (1,565)	983 (895)	757 (932)	0.107
Transfusion requirements					
PRBC	48 (27)	14 (25)	18 (33)	16 (25)	0.811
FFP	7 (4.0)	3 (5.3)	0 (0.0)	4 (6.3)	
Platelets	4 (2.3)	1 (1.8)	3 (5.4)	0 (0.0)	
Cell saver	1 (0.6)	0.0 (0.0)	0 (0.0)	1 (1.6)	

Values given as *n* (%) unless otherwise stated. n, number of patients; SD, standard deviation; PRBC, packed red blood cells; FFP, fresh frozen plasma.

Bold font indicates statistical significance.

Staged aortic repair was performed in 16% of the patients.

The mean operating time declined significantly between early and recent phases (321 vs. 241 min; *p* = 0.002).

A significant reduction of used contrast media was observed when comparing early and recent phases (398 vs. 190 mL; *p* = 0.001).

With an average of 65 min, total fluoroscopy time remained stable throughout the observed period.

### Outcomes and follow-up

Overall, in-hospital mortality was 4.5% and showed a non-significant trend (*p* = 0.062) favoring the recent treatment phase (1.6%) compared to the intermediate and the early phases (3.6 and 8.8%). When comparing the early with the recent treatment phases, length of ICU stay declined significantly (8.1 vs. 2.7 days; *p* < 0.001) and length of hospital stay showed a non-significant downward trend (20 vs. 15 days; *p* = 0.248).

Any MAE occurred in 34% of the cohort. Comparing early and recent phases, the occurrence of acute kidney injury (16 vs. 4.7%; *p* = 0.034) and respiratory failure (18% vs. 0%; *p* = 0.001) declined significantly.

Paraplegia showed a non-significant downward trend when comparing the three treatment phases (8.8 vs. 3.6 vs. 1.6%; *p* = 0.062).

Among perioperative endoleaks, type II represented the most common entity (20%), followed by type III (11%) and type I (8%). No significant changes were observed between time periods.

### Univariate analyses

Results of the univariate analyses are given in Table 4. The univariate analysis showed a non-significant association between the operation being performed during the recent treatment phase compared to the early phase and in-hospital death (OR 0.17; 95% CI 0.02–1.46). For age and sex, no associations were found with regard to the primary outcome event.

**Table 4:** Univariate analysis: associations of different variables with in-hospital death.

Variable	*n*/*N* (%)	OR	95% CI	*p*-value
Time of operation				
Early phase	5/57 (8.8)	Ref.		
Intermediate phase	2/55 (3.6)	0.39	0.05–1.91	0.276
Recent phase	1/64 (1.6)	0.17	0.02–1.46	0.105
Age				
Age > median	4/87 (4.6)	1.02	0.24–4.46	0.974
Age ≤ median	4/89 (4.5)	Ref.		
Sex				
Male sex	5/140 (3.6)	0.41	0.10–2.08	0.235
Female sex	3/36 (8.3)	Ref.		
Type of procedure				
Emergency	3/23 (13)	4.40	0.86–19.5	0.052
None-emergency	5/153 (3.3)	Ref.		
Aortic aneurysm diameter				
Diameter > median	2/77 (2.6)	0.41	0.06–1.83	0.282
Diameter ≤ median	6/98 (6.1)	Ref.		
Type of aortic pathology				
Aortic dissection *n*, (%)				
No	8/171 (4.7)	Ref.		
Yes	0/5 (0.0)	1.75	0.01–17.7	0.731
Aneurysm Type				
TAAA Crawford Type I/II/III/V	3/40 (7.5)	2.09	0.41–8.93	0.327
Juxta/pararenal AAA/ Crawford Type IV	5/131 (3.7)	Ref.		
Comorbidities				
Hypertension				
No	0/5 (0.0)	Ref.		
Yes	8/171 (4.7)	0.57	0.06–77.4	0.731
Coronary artery disease				
No	5/83 (6.02)	Ref.		
Yes	3/93 (3.23)	0.52	0.10–2.19	0.381
COPD				
No	6/143 (4.20)	Ref.		
Yes	2/33 (6.06)	1.47	0.21–6.75	0.645
Smoking (past or active)				
No	1/43 (2.33)	Ref.		
Yes	7/133 (5.26)	2.33	0.40–44.3	0.434
Diabetes				
No	8/139 (5.76)	Ref.		
Yes	0/37 (0.0)	0.021	0.002–1.72	0.176
Cancer				
No	6/130 (4.62)	Ref.		
Yes	2/46 (4.35)	0.94	0.13–4.25	0.940
ASA-Classification				
II&III	7/163 (4.29)	Ref.		
IV	1/13 (7.69)	1.86	0.10–11.7	0.577
Type of device				
Custom made device	5/125 (4.00)	0.67	0.16–3.35	0.589
Off-the-shelf device	3/51 (5.88)	Ref.		
Vessels incorporated				
4	5/142 (3.52)	0.38	0.09–1.92	0.198
<4	3/34 (8.82)	Ref.		
Operating time (per 10 min increase)	27 (13)	1.06	1.02–1.11	**0**.**003**
Occurrence of MAE^a^				
No	0/115 (0.0)	Ref.		
Yes	8/60 (13.3)	37.40	4.52–4,869	**<0**.**001**

*n*, number of patients; OR, odds ratio; CI, confidence interval; TAAA, thoracoabdominal aortic aneurysm; AAA abdominal aortic aneurysm; COPD, chronic obstructive pulmonary disease; ASA, American Society of Anesthesiologists; MAE, major adverse event.

a Combined outcome comprising blood loss >1,000 mL, myocardial infarction, acute kidney injury (i.e., >50% decrease in glomerular filtration rate), respiratory failure, paraplegia, stroke, bowel ischemia. Bold values indicate statistical significance.

Emergency procedures showed a non-significant trend towards higher in-hospital death compared to non-emergency procedures (OR 4.40; 95% CI 0.86–19.5).

Equally, presence of TAAA Crawford types I/II/III/V were associated with a non-significant trend towards higher in-hospital death rates compared to juxtarenal, pararenal AAAs or TAAAs Crawford type IV (OR 2.09; 95% CI 0.41–8.93).

None of the assessed comorbidities showed a significant association with the occurrence of the primary outcome event.

An elevated operating time was significantly associated with in-hospital mortality (per 10 min increase; OR 1.06; 95% CI 1.02–1.11).

The occurrence of a MAE was significantly associated with in-hospital mortality (OR 37.40; 95% CI 4.52–4,869).

### Follow-up

The mean FU was 19.4 months (590 days; Table 5).

**Table 5:** Outcomes and follow-up.

Variable	Overall (*n* = 176)	Early phase (<2017) (*n* = 57)	Intermediate phase (*n* = 55)	Recent phase (>2020) (*n* = 64)	*p*-value
In-hospital outcomes					
Length of ICU stay in days (mean; SD)	4.7 (9.1)	8.1 (11)	3.5 (5.6)	2.7 (8.4)	**<0**.**001**
Length of hospital stay in days (mean; SD)	17 (15)	20 (18)	16 (13)	15 (12)	0.248
In-hospital death	8 (4.5)	5 (8.8)	2 (3.6)	1 (1.6)	0.062
Any secondary intervention	78 (44)	31 (54)	22 (40)	25 (39)	0.095
Aortic secondary intervention	34 (19)	11 (19)	10 (18)	13 (20)	0.881
Any MAE	60 (34)	21 (37)	23 (42)	16 (25)	0.174
Estimated blood loss >1,000 mL	29 (16)	6 (11)	15 (27)	8 (13)	0.805
Myocardial infarction	7 (4.0)	2 (3.5)	3 (5.5)	2 (3.1)	0.911
Acute kidney injury^a^	15 (8.5)	9 (16)	3 (5.5)	3 (4.7)	**0**.**034**
Respiratory failure	17 (9.7)	10 (18)	7 (13)	0 (0.0)	**0**.**001**
Paraplegia	8 (4.5)	5 (8.8)	2 (3.6)	1 (1.6)	0.062
Stroke	11 (6.3)	5 (8.8)	1 (1.8)	5 (7.8)	0.887
Bowel ischemia	4 (2.3)	2 (3.5)	1 (1.8)	1 (1.6)	0.487
Endoleak					
Endoleak Type I	14 (8.0)	4 (7.0)	3 (5.5)	7 (11)	0.489
Endoleak Type II	36 (20)	14 (25)	12 (22)	10 (16)	
Endoleak Type III	19 (11)	9 (16)	2 (3.6)	8 (13)	
Follow-up					
Follow-up in days (mean; SD)	590 (735)	827 (960)	761 (703)	233 (233)	**<0**.**001**
Follow-up in years (mean; SD)	1.62 (2.01)	2.27 (2.63)	2.08 (1.93)	0.64 (0.64)	**<0**.**001**
Aortic secondary intervention	41 (23)	16 (28)	11 (20)	14 (22)	0.434
Death within 30 days	8 (4.5)	4 (7.0)	3 (5.5)	1 (1.6)	0.147
Death during follow-up	16 (9.1)	8 (14)	7 (13)	1 (1.6)	**0**.**016**

Values given as *n* (%) unless otherwise stated. n, number of patients; SD, standard deviation; ICU, intensive care unit; MAE, major adverse event.

a Defined as >50% decrease in glomerular filtration rate.

Bold font indicates statistical significance.

**Figure 2:**
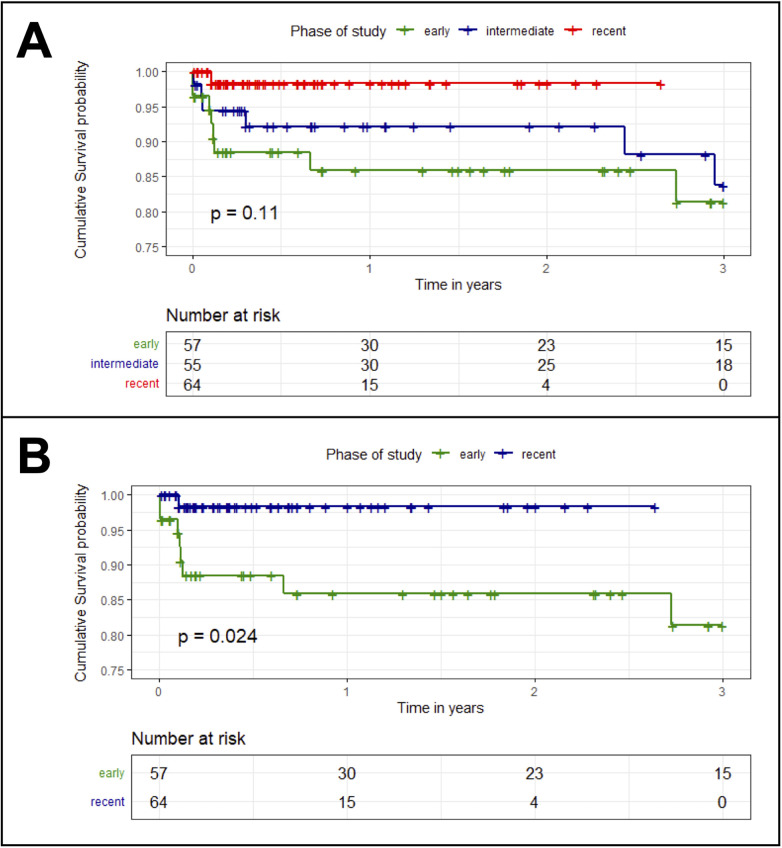
Kaplan–meier survival curves comparing patients treated in all three phases **(A)** and patients treated in early phase to those treated in recent phase **(B)** early phase = operation between 2007 and 2016; intermediate phase = operation between 2017 and 2020; recent phase = operation between 2021 and 2023.

The Kaplan–Meier curves are given in Figure 2. Probability of survival was highest in the recent treatment phase and lowest in the early phase. The difference between the recent and the early phase was statistically significant (*p* = 0.024).

The univariate analyses (Table 6) revealed, that aneurysm type (TAAA Crawford types I/II/III/V vs. juxta/pararenal AAA or TAAA Crawford type IV) was significantly associated with mortality during FU (OR 3.94; 95% CI 1.35–12). Aortic dissection showed a trend towards higher mortality during FU (OR 2.60; 95% CI 0.13–19).

**Table 6:** Univariate analysis: associations of different variables with death during follow-up.

Variable	*n*/*N* (%)	OR	95% CI	*p*-value
Age				
Age > median	8/87 (9.2)	1.03	0.36–2.92	0.962
Age ≤median	8/89 (9.0)	Ref.		
Sex				
Male sex	11/140 (7.9)	0.53	0.18–1.78	0.268
Female sex	5/36 (14)	Ref.		
Type of procedure				
Emergency	4/23 (17)	2.5	0.64–7.96	0.149
None-emergency	12/153 (7.8)	Ref.		
Aortic aneurysm diameter				
Diameter > median	6/77 (7.8)	0.74	0.24–2.10	0.584
Diameter ≤median	10/98 (10)	Ref.		
Type of aortic pathology				
Aortic dissection *n*,(%)				
No	15/171 (8.8)	Ref.		
Yes	1/5 (20)	2.60	0.13–19	0.406
Aneurysm Type				
TAAA Crawford Type I/II/III/V	8/40 (20)	3.94	1.35–12	**0**.**011**
Juxta/ pararenal AAA/Crawford Type IV	8/131 (6.0)	Ref.		
Comorbidities				
Hypertension				
No	0/5 (0.0)	Ref.		
Yes	16/171 (9.4)	1.17	0.12–156	0.916
Coronary artery disease				
No	9/83 (11)	Ref.		
Yes	7/93 (7.5)	0.67	0.23–1.88	0.447
COPD				
No	11/143 (7.7)	Ref.		
Yes	5/33 (15)	2.14	0.63–6.41	0.187
Smoking (past or active)				
No	3/43 (7.0)	Ref.		
Yes	13/133 (9.8)	1.44	0.44–6.53	0.581
Diabetes				
No	16/139 (12)	Ref.		
Yes	0/37 (0.0)	0.10	0.001–0.77	**0**.**021**
Cancer				
No	14/130 (11)	Ref.		
Yes	2/46 (4.4)	0.38	0.06–1.42	0.208
Complications				
Acute kidney injury				
No	10/160 (6.3)	Ref.		
Yes	6/15 (40)	10.0	2.88–34.0	**<0**.**001**
Respiratory failure				
No	12/158 (7.6)	Ref.		
Yes	4/17 (24)	3.74	1.00–13.0	**0**.**041**
Stroke				
No	13/164 (7.9)	Ref.		
Yes	3/11 (27)	4.36	1.03–18.4	**0**.**046**
Bowel ischemia				
No	15/171 (8.8)	Ref.		
Yes	1/4 (25)	3.47	0.17–29	0.295
Myocardial infarction				
No	14/168 (8.3)	Ref.		
Yes	2/7 (29)	4.40	0.59–23	0.093
ASA-classification				
II&III	13/163 (8.0)	Ref.		
IV	3/13 (23)	3.46	0.71–13	0.084
Type of device				
Custom made device	10/125 (8.0)	0.65	0.23–2.0	0.433
Off-the-shelf device	6/51 (12)	Ref.		
Vessels incorporated				
4	10/142 (7.0)	0.35	0.12–1.1	0.062
<4	6/34 (18)	Ref.		
Operating time (per 10 min increase)	27 (13)	1.05	1.02–1.09	**0**.**002**
Occurrence of MAE^a^				
No	4/115 (3.5)	Ref.		
Yes	12/60 (20)	6.94	2.29–26	**0**.**001**

*n*, number of patients; OR, odds ratio; CI, confidence interval; TAAA, thoracoabdominal aortic aneurysm; AAA abdominal aortic aneurysm; COPD, chronic obstructive pulmonary disease; ASA, American Society of Anesthesiologists; MAE, major adverse event.

a Combined outcome comprising blood loss >1,000 mL, myocardial infarction, acute kidney injury (i.e., >50% decrease in glomerular filtration rate), respiratory failure, paraplegia, stroke, bowel ischemia. Bold values indicate statistical significance.

None of the comorbidities showed a significant association with mortality during FU.

The occurrence of a MAE was significantly associated with death during FU (OR 6.94; 95% CI 2.29–26). Regarding the different components of the combined outcome MAE, perioperative acute kidney injury (OR 10.0; 95% CI 2.88–34.0), respiratory failure (OR 3.74; 95% CI 1.00–13.0), and stroke (OR 4.36; 95% CI 1.03–18.4) were significantly associated with mortality during FU.

## Discussion

This retrospective analysis demonstrates a continuous evolution in patient selection, procedural techniques, and clinical outcomes in the endovascular repair of TAAA over a 17-year period.

One of the key findings was the marked reduction in perioperative morbidity, reflected by a significant decline in ICU stay from 8.1 to 2.7 days and by significant reductions of renal and respiratory complications. Declining rates of renal and respiratory complications over time were also found in a large retrospective study of data from ten prospective, nonrandomized, device exemption studies from 2015 to 2023. (12) The reason for the lower rate of perioperative acute renal failure remains unclear. Previous studies on patients undergoing endovascular repair of complex aortic aneurysms detected an association between the amount of contrast media and the occurrence of perioperative renal failure (13, 14). Despite a significant reduction of contrast media use (398 vs. 190 mL in early vs. recent period) over the study period, our data did not show an association between the amount of contrast media and the occurrence of acute kidney injury (per 10 mL increase; OR 0.99; 95% CI 0.99–1.99; *p* = 0.922). A similar reduction of contrast media throughout the learning curve was observed by a prospective single center study including 50 patients who underwent f/bEVAR between 2014 and 2017. While a mean of 157 mL were used to treat the earliest cohort of patients, the most recent cohort was treated by using 108 mL of contrast media (*p* = 0.028) (15). Another finding of our study was a non-significant trend towards a lower paraplegia rate in the recent phase (*p* = 0.062). Besides a more meticulous adherence to SCI prevention strategies towards the end of the study period, this finding might be traced back to a higher percentage of j/pAAAs in the recent phase (72%) compared to the early (63%) and intermediate (67%) phases, which are generally at lower risk of SCI compared to TAAAs (16). Other observations from our study were confirmed including significant reductions in operating time (452 vs. 362 min) and fluoroscopy time (130 vs. 99 min) and trends towards shorter ICU stay (4 vs. 2 days) and length of hospital stay (7 vs. 5 days) (15). In general, the reduced perioperative morbidity mirrors advancements in perioperative management, refined anesthetic management, better intraoperative organ protection strategies, and improved patient selection.

Although not statistically significant, the study showed a trend towards lower in-hospital mortality over time. Declining mortality (trends) over the past years was found by previous studies (12, 17). This finding might be predominantly caused by an increasing experience of treating surgeons and departments with complex f/bEVAR procedures. A volume outcome relation for complex endovascular procedures was demonstrated before (18). Furthermore, it is likely that a generational change of vascular surgeons has occurred over the past years. As endovascular techniques have been evolving and developed only over the past few decades, elder generations of vascular surgeons typically acquired endovascular skills at a later stage in their careers. With the implementation of dedicated endovascular trainings and certifications, younger generations of vascular surgeons might experience steeper learning curves and be more familiar with endovascular procedures compared to elder generations. Temporal improvements may partly reflect changes in patient selection and device technology rather than purely procedural learning.

Another important finding of this study was the significant increase in complexity of endografts incorporating significantly more vessels over time. The increasing number of incorporated vessels per patient was found by other studies (17, 19–21). It may be traced back to an increased anatomical complexity over time, but also to a higher awareness of disease progression with four-vessel fenestrated endografts being easier to extend proximally compared to endografts landing within the visceral segment (22). Despite a rise in the average number of target vessels and a shift from scallops to fenestrations, procedure times and contrast agent volumes were significantly reduced. This likely reflects advances in imaging technology, such as high-resolution CTA, and fusion-based intraoperative navigation. However, this progress was accompanied by higher radiation exposure, likely due to longer fluoroscopy times associated with complex procedures.

Device selection also evolved throughout the study period. While fenestrated endografts with or without scallop manufactured by Cook (Cook Medical, Bloomington, USA) represented the predominant device type in the early phase, over time we observed a decline of endografts with scallops and those with less than four fenestrations. This downward movement happened in favor of an increased employment of the fenestrated Anaconda stent graft (Vascutek/Terumo Aortic, Inchinnan, UK). This shift may reflect advantages in challenging aortic anatomies, improved familiarity among operators, or institutional procurement policies. While fenestrated endografts are usually employed to treat j/pAAAs, TAAAs regularly require branched endografts or combinations of branches and fenestrations. The implantation rate of the t-Branch multibranched stent graft remained stable throughout the study period. Due to its off-the-shelf availability, this device is commonly used in urgent cases and in elective cases in our institution. However, if the aneurysm does not involve the entire pararenal segment of the aorta (e.g., TAAA Crawford type V) and time allows planning of a CMD, in recent years we preferred employment of endografts with combinations of branches and fenestrations or combinations of outer branches and inner branches.

Importantly, our univariate analysis demonstrated longer procedure time to be associated with perioperative mortality. Although the retrospective study design may be subject to confounders and/or reversed causation, the finding is in line with prior research (23).

Equally, the occurrence of a MAE was highly significantly associated with perioperative mortality. Comparably, a retrospective analysis of 596 patients treated in four Italian centres with f/bEVAR identified the occurrence of any postoperative complication to be associated with perioperative mortality (24). Equally, urgent setting of f/bEVAR was associated with perioperative mortality (*p* = 0.001) (24). In the present study, emergency procedures showed a non-significant trend towards higher mortality (*p* = 0.052). The difference in effect sizes might be explained by the lower numbers of overall patients and particularly of emergency patients in our study.

The occurrence of MAEs was strongly associated with mortality during follow-up, underscoring the need for meticulous intraoperative management and complication prevention. In detail, acute kidney injury, respiratory failure, and stroke were significantly associated with mortality during follow-up. This conforms in part to the results of the previously mentioned retrospective study of patients treated in four Italian centres, which proved that cardiac and pulmonary complications, as well as bowel ischemia and spinal cord ischemia were associated with death during follow-up (24). Another similarity in results of both studies is, that the extent of TAAAs was associated with death during follow-up (24). Moreover, the present study showed that increased operating time was associated with mortality during follow-up. A longer operating time is likely to be a surrogate marker for higher complexity of f/bEVAR or the occurrence of intraoperative complications and therefore might affect short- and long-term outcomes.

In contrast to earlier reports (24, 25), common comorbidities —including chronic renal failure, diabetes, hypertension, coronary artery disease, and COPD— were not significantly associated with mortality in the present study.

Overall, these results indicate that f/bEVAR for complex aortic aneurysms has become safer and more effective over time, despite increasing procedural complexity and patient age. A generational change of vascular surgenons, continued advances in technique with the implementation of high-performing hybrid operating rooms, image fusion, further developments in device design, strict adherence to SCI prevention protocols, and long-term surveillance are possible reasons for improved outcomes.

## Limitations

This retrospective, single-center study is subject to inherent limitations, including limited generalizability and the inability to establish causality. Variability in follow-up adherence may have introduced selection bias, particularly affecting the assessment of long-term outcomes. Data collection by treating physicians raises the possibility of underreporting complications, though this bias is likely non-differential.

No multivariable regression analysis was performed due to a low patient number, so observed associations may be influenced by unmeasured confounders or reversed causation. Furthermore, patient-centered outcomes and precise causes of death were not available, limiting interpretation beyond clinical endpoints.

## Conclusions

This study demonstrates a clear improvement in clinical outcomes and procedural strategies for f/bEVAR in TAAA over nearly two decades. The observed reduction in perioperative complications, ICU stay, and in-hospital mortality reflects advances in patient selection, imaging technology, and operator experience.

While endovascular repair has become an increasingly safe and established approach for complex TAAA, continued development, particularly in image-guided and AI-assisted planning, and structured long-term follow-up are essential to further optimize outcomes.

## Data Availability

The raw data supporting the conclusions of this article will be made available by the authors, without undue reservation.
